# A Longitudinal Examination of Developmental Covariates of Sexual Behavior Problems among Youth Referred to Child Protection Services

**DOI:** 10.1177/10790632211047184

**Published:** 2021-09-30

**Authors:** Stéphanie Chouinard-Thivierge, Patrick Lussier, Isabelle V. Daignault

**Affiliations:** 1 School of Social Work and Criminology, 4440 Université Laval, Québec, Canada; 2 Centre International de Criminologie Comparée (CICC), Montréal, Canada; 3 Centre de Recherche Universitaire sur les Jeunes et les Familles (CRUJeF), Québec, Canada; 4 School of Criminology, 5622 Université de Montréal, Montréal, Canada

**Keywords:** child adversity, child protection services, longitudinal study, sexual, behavior problem, development factors

## Abstract

Little is known about the development of childhood sexual behavior problems (SBP) in terms of continuity and discontinuity into adolescence. Prior studies have espoused a nondevelopmental approach focusing on the clinical profiles of these youths at the time of their referral. To address this gap, the current study proposes an examination of the developmental covariates involved in the continuity of SBP among a sample of 340 children and adolescents referred to Child Protection Services (CPS) in Quebec, Canada. Children’s CPS contacts from birth up to age 17 were inspected, allowing to recreate the life history of social and familial adversities during that period. Logistic regression models were performed and helped to identify developmental covariates of childhood-onset SBP and its persistence into adolescence. Findings suggest that children with childhood-onset SBP that persisted into adolescence have experienced various life adversities. The study findings provide some preliminary evidence of the developmental pathways of SBP.

## A Longitudinal Examination of Developmental Covariates of Sexual Behavior Problems among Youth Referred to Child Protection Services

Clinical studies on the effects of child sexual abuse has led to research on sexual behavior problems (SBP) among children and adolescents (e.g., Friedrich et al., 1991), namely sexual behaviors that are developmentally inappropriate, repetitive, intrusive, coercive, and/or aggressive (Chaffin et al., 2000; Lussier et al., 2017; Pithers et al., 1998). For the most part, this growing body of research has been descriptive, concerned with identifying individual- and familial-level characteristics of youth with SBP (e.g., Lussier & Healey, 2010). Clinical profiles of youth with SBP show that sexual victimization is part of the developmental antecedents for some but as a group their developmental history is more complex and extremely heterogeneous (e.g., Lussier et al., 2019; Wanklyn et al., 2012). These observations have led researchers to argue for a developmental approach that focuses on the description and explanation of the origin and development of SBP (Chaffin et al., 2000; Elkovitch et al., 2009; Lussier, 2017). The need for a developmental approach is further supported by empirical observations that suggest that SBP have a distinct developmental course in different individuals. For example, the age of onset of SBP varies considerably, with some youth showing childhood-onset and others adolescence-onset (e.g., Curwen et al., 2014; Vizard, Hickey, French, & McCrory, 2007). The developmental course is sufficiently heterogeneous that recent longitudinal studies have found both continuity and discontinuity of childhood-onset SBP into adolescence (e.g., Friedrich et al., 2005; Grossi et al., 2017). These observations demonstrate the need for more thorough investigation of risk factors for the onset of SBP, particularly as a preface to developing more appropriate intervention and prevention methods (Lussier & Chouinard-Thivierge, 2017). Until now, studies have been primarily clinical and nondevelopmental, making it difficult to draw firm conclusions about continuity/discontinuity of SBP from childhood to adolescence and the developmental antecedents that characterize the context in which it occurs.

## Sexual Behavior Problems in Childhood and Adolescence

Epidemiological and clinical research has shown that some sexually abused children show evidence of SBP (e.g., Friedrich et al., 1992). These observations led to the examination of the link between experience of sexual victimization, its context, and the presence of SBP among children and adolescents (e.g., Aebi et al., 2015; Burton et al., 1997; Salter et al., 2003). However, sexual victimization has not been found to be a prerequisite for the onset of SBP (e.g., Silovsky & Niec, 2002), suggesting that other developmental factors are also involved. This finding led to questions about the nature and timing of developmental factors, such as the quality of a child’s family environment and experiences of maltreatment. Various familial, contextual, and individual factors have been correlated with SBP in childhood and adolescence and numerous studies have shown that the lives of those with SBP are characterized by multiple instances of adversity (Bladon et al., 2005; Gray et al., 1999; Hall et al., 2018; Leon et al., 2008; Letourneau et al., 2004; Lussier et al., 2019; Merrick et al., 2008; Szanto et al., 2012; Wieckowski et al., 1998). For example, a study conducted by Vizard, Hickey, French, & McCrory, (2007) showed that various factors related to family environment–exposure to intimate partner violence, inconsistent parenting, lack of parental supervision, lax sexual boundaries, and physical neglect–were commonly observed among youth with SBP and most were correlated with SBP that developed early in childhood (i.e., before 10 years of age) and continued into adolescence.

Studies have also found that maltreatment (e.g., physical and psychological abuse) is correlated with the development of SBP (e.g., Lussier et al., 2019; Tarren-Sweeney, 2008) and the age at which children experience maltreatment has been identified as relevant (Merrick et al., 2008). Studies have shown that youth with SBP also tend to exhibit nonsexual behavior problems such as aggression, problematic drug and/or alcohol use, delinquency, or self-harming (Friedrich et al., 1992; Letourneau et al., 2004; Vizard, Hickey, French, & McCrory, 2007). Youth experiencing various life adversities and behavior problems are often placed in out-of-home living environments to ensure their safety, a form of placement that has been associated with the development of SBP (Grossi et al., 2017; Hall et al., 2018; Vizard, Hickey, French, & McCrory, 2007). These studies highlight the need to broaden the scope of investigation in factors involved in the development of SBP.

Although researchers have suggested that a development perspective is a useful way to better understand SBP (e.g., Chaffin et al., 2000; Elkovitch et al., 2009), there has been little empirical research on its development or potential developmental covariates. The literature in this regard is largely retrospective and based on samples of convicted adult offenders (e.g., Jespersen et al., 2009). These findings, despite their limitations, suggest the importance of examining the role of experiences of sexual abuse on SBP in the broader context of adverse childhood experiences ([ACE; Felitti et al., 1998], e.g., Barra et al., 2018; Drury et al., 2017; Grady et al., 2017; Kahn et al., 2020; Levenson et al., 2016). A longitudinal research design is needed to capture the evolution of SBP over time, taking into account age-graded risk and protective factors (e.g., Lussier, 2017). The few previous developmentally oriented longitudinal studies have led to the identification of distinct developmental patterns of SBP among youth, especially in terms of the continuity and discontinuity of SBP in childhood and adolescence (Friedrich et al., 2005; Grossi et al., 2017; McCrory et al., 2008). More precisely, findings show that only a small number of children with SBP exhibit a persistent pattern of SBP into adolescence (see also Lussier et al., 2019; Vizard, Hickey, French, & McCrory, 2007). Furthermore, these studies show that patterns of SBP are associated with different development factors, suggesting that varying methods of intervention are needed (e.g., McCrory et al., 2008; Vizard, Hickey, & McCrory, 2007). For example, prior studies suggest that childhood-onset SBP is more likely to be associated with adversity in early life, such as parental neglect and abuse, than is the case for adolescence-onset SBP (Lussier et al., 2019).

Most previous studies of SBP have been guided by theoretical frameworks that focus on the etiological role of sexual victimization. In contrast, more recent empirical findings from longitudinal studies have shown that a comprehensive understanding of the development of SBP among youth requires investigation of a broader range of adversities in early life and nonsexual behavior problems. While prior research has focused on adolescence, the present study focuses on earlier developmental stages and, rather than relying on previous theoretical frameworks that stress the importance of a sexual dimension, is guided by a developmental perspective. The current study explores particularly the statistical associations between childhood life adversities, nonsexual behavior problems, and childhood-onset and continuity of SBP into adolescence.

## Aim of the Study

Attachment theories related to sexual offending in adolescence have stressed the role and importance of ACE other than sexual abuse. Attachment bonds between parents and children are understood to be pivotal because they provide the foundation for the child’s internal representations of self and others, as well as the basis for future social interactions (e.g., Marshall, 1989; Marshall et al., 1993). Poor parenting (e.g., neglect, abuse, and violence) and disruptions in parenting early in a child’s development can result in insecure attachment bonds (e.g., Grady et al., 2017; Smallbone, 2006; Smallbone & Dadds, 2000) that can negatively influence children’s social interactions, leading to difficulties that may affect their sociosexual development after puberty (e.g., Marshall & Barbaree, 1990). While attachment theorists have not emphasized the role of childhood-onset SBP in their studies of the origins and development of sexual offending in adolescence, prior studies have shown that childhood-onset SBP are likely to occur concurrently with other behavior problems and with experiences of adversity in early life (e.g., Grossi et al., 2017; Hall et al., 2018; McCrory et al., 2008). In line with these previous empirical findings, the focus of the current study is on the developmental covariates of childhood-onset SBP and its persistence into adolescence. The study is theoretically framed around a developmental perspective of sexual offending, especially the role and importance of attachment within the context of human development, and a set of covariates—ACE—are examined, grounded in the theoretical approach of attachment theory. The current study looks at individual and contextual developmental antecedents (i.e., family environment, history of victimization, and behavior problems) in a retrospective-longitudinal examination of childhood-onset SBP and its persistence into adolescence. Early life course and exposure to adversity in infancy, early childhood, and late childhood were systematically examined in a sample of children who had been referred to Child Protection Services in Quebec, with the role and importance of the timing of the occurrence of these factors taken into account. This retrospective-longitudinal examination of covariates from infancy to late childhood makes it possible to identify significant developmental covariates in the persistence of childhood-onset SBP into adolescence.

## Method

### Sample

This research deals with children and adolescents referred to the Child Protection Services (CPS) of the Centre Intégré Universitaire de Santé et de Services Sociaux (CIUSSS) de la Capitale-Nationale in Quebec, Canada. Referrals made to the CPS are analyzed by authorized professionals; if they decide that the development or security of the child or adolescent is being compromised based on regulations laid out in section 38 of the Quebec Youth Protection Act, CPS provide services in order to end the situation that endangers their development or security, and to prevent its recurrence. Data used in this study were retrieved from the files of children and adolescents whose CPS files indicated at least one occurrence of SBP before the age of 18. The complete file as well as information on the services offered by CPS (e.g., referral, investigation, and intervention) were considered. A total of 1933 cases were identified for the period between 2002 and 2016.

Given that resources were insufficient to permit detailed examination of all 1933 cases, a subsample of this group was subjected to detailed analysis to identify the various adversities involved based on data from the CPS computerized case-processing system. A stratified sampling approach was used to identify this subgroup. Because adolescents made up 68% of the total sample and were therefore overrepresented, participants were divided into two groups based on the estimated age of onset of SBP. The childhood-onset group was composed of those whose SBP had been identified before they reached the age of 12; for those in the adolescence-onset group, SBP had been identified during ages 12–17. To avoid the presence of right-censured data (e.g., a child who was 11 years old when the data was retrieved), the stratified sample was composed of only those who were at least 17 years old at the time the data were collected. With these criteria in mind, we randomly selected a subsample of youth with childhood-onset SBP and a subsample of youth with adolescence-onset SBP.

The resources available (e.g., time and number of research assistants available to code the files) made it possible to conduct a detailed longitudinal and chronological examination of CPS files for almost 20% of the children in the total sample. The present study is therefore based on a subsample of 340 individuals who showed evidence of SBP prior to turning 18 and had at least one contact with CPS during that time. The stratified sampling approach led to the identification of a subsample of 158 cases of childhood-onset SBP (46.5%) and a subsample of 182 cases of adolescence-onset SBP (53.5%). Statistical analyses, including logistic regression analyses, were performed to determine whether both groups were representative of their respective groups in the full sample^1^. Despite the random selection of cases for each group, some statistically significant differences were found. Analyses showed that the childhood-onset subsample tended to have had more CPS referrals than those in the total sample, while the first referral to CPS for those in the adolescence-onset group had been at an earlier age than for those in the total sample. To account for these relatively minor, but statistically significant discrepancies, statistical controls were used in the current study (e.g., total number of CPS referrals and age at first CPS referral). Descriptive information about this sample is presented in Table 1. The sample was composed of 68.5% boys (*n* = 233) and 31.5% girls (*n* = 107), and the large majority were White (95.2%). With regard to the history of CPS referrals (for reasons specified under section 38 of the Youth Protection Act), the first referral for about one in three (31.5%) had occurred before the age of 6 years.

**Table 1.** Descriptive Information about the Sample.

Variables		Descriptive Statistics	
	Categories	%	Mean (*S.D.*)
Sex	Male	68.5	
	Female	31.5	
Ethnicity	White	95.2	
	Other	4.8	
Number of siblings			1.9 (1.4)
Parental criminal record		28.8	
History of Child Protection Service (CPS) referrals			
Early age at 1^st^ referral (i.e., before 6 years old)		31.5	
Total number of referrals			7.5 (6.1)
	1	10.9	
	2–3	19.2	
	4–9	40.1	
	10 or more	29.8	
History of sexual behavior problems (SBP)			
Age at first SBP occurrence			11.7 (2.8)
	0–5 y.o.	1.2	
	6–11 y.o.	45.3	
	12 and older	53.5	
Total number of occurrences of SBP			1.8 (1.2)
	1	58.5	
	2	23.8	
	3 or more	17.7	

*Note*. Sample size varies between 292 and 340 due to missing information

### Procedures

CPS files include information provided by authorized professionals (e.g., social workers) following a child’s first referral to CPS. The files examined for this study were not limited to instances where children and adolescents were referred to CPS for SBP. The entire CPS file was therefore examined and systematically coded for each participant, making it possible to recreate the entire history of reported adversity and behavioral problems during childhood and adolescence. Information was gathered systematically and chronologically by examining the entire history of contact with CPS from birth to age 17. Although this procedure is not the equivalent of a prospective longitudinal study with repeated measurements over time, the research design makes it possible to fill a gap in the scientific literature by examining the developmental context for the youth in our subsample.

A coding scheme was developed using a life history calendar approach and was specifically designed to collect longitudinal information regarding the child’s family environment, history of victimization, behavioral problems, and SBP. This coding scheme made it possible to document the presence of various covariates across three different developmental periods in childhood—(a) infancy (before the age of two), (b) early childhood or preschool years (from two to 5 years old), and (c) late childhood or school entry and elementary school years (from 6 to 11 years old)—and in adolescence (from 12 to 17 years old inclusive). Information about the developmental periods of infancy, early, and late childhood made it possible to explore the developmental covariates for childhood-onset SBP and its continuity into adolescence. Because the focus is on childhood, adolescence covariates were not included in the current study. Analyses were conducted to test for interrater reliability in using the coding scheme, based on two raters and a randomly selected subsample of 25 cases. For categorical variables, Kappa coefficients ranged from 0.79 to 1.00, while for continuous variables, intraclass correlation coefficients ranged from 0.99 to 1.00.

## Measures

The current study was based on examination of the CPS files of each participant from birth to age 17 and was not limited to examination of files in which the referral to CPS was based on SBP. Youth, especially children with SBP, are likely to have been in contact with CPS both prior to and after referral for SBP for various other reasons, such as physical abuse and neglect (Lussier et al., 2019). The children in our sample had been referred to CPS many times throughout their childhood and adolescence: a minority (10.9%) had only one referral but almost one-third of the sample (29.8%) had 10 or more separate CPS referrals. Adversities noted in the files were coded from birth to late childhood, while reports of SBP were coded from birth to age 17. The data below are therefore restricted to the adversities and instances of SBP that came to the attention of CPS.

*Control variables.* A series of sociodemographic and CPS indicators were used as statistical control variables. Sociodemographic indicators include sex (0 = female; 1 = male), ethnicity (0 = White; 1 = other), and number of siblings. Indicators about CPS contacts were (a) an early age at the first CPS referral, that is, before the age of six (0 = no; 1 = yes); (b) parental criminal record (0 = no; 1 = yes); and (c) total number of childhood CPS referrals (for reasons related to section 38 of the Youth Protection Act). These three indicators were used as control variables as they suggest that files containing them may have more information about the familial environment during the period under study: repeated contact with the family and with the child from an early age can be expected to lead to increased familiarity with the case. Although these indicators are concerned with the environment of the youth in the sample during the period under study, they also make it possible to control for the availability of different levels of information for research participants.

*Childhood familial environment.* Several indicators related to familial environment were investigated for each of the three developmental periods. These included whether a parent had a drug and/or alcohol abuse problem as assessed by CPS and whether there had been a disruption in the familial environment due to the long-term absence (i.e., more than a year without contact due to abandonment, incarceration, hospitalization, or death) of at least one biological parent. Inappropriate parental behaviors that required CPS intervention were also recorded. These included: (a) association with antisocial peers (e.g., association with individuals involved in criminal activities); (b) impulsive behavior and/or instability of the familial environment (e.g., inconsistent discipline or attitude toward the child, interpersonal instability, and frequent moves/changes of residence or work); (c) child’s exposure to intimate partner violence; (d) inadequate or inappropriate sexual boundaries (e.g., watching pornography or sexual contact in the presence of the child); and (e) parental neglect (e.g., failing to meet the child’s food, hygiene, or health needs). Placement outside the home (i.e., in an alternative living environment such as a foster family, with a relative, or in a group home or rehabilitation center) was also considered. These variables were coded as either absent or present based on information in the file (0 = absence, 1 = presence).

*History of abuse.* Examination of CPS files also made it possible to code for various indicators of victimization (whether intrafamilial or extrafamilial) experienced in each of the three developmental periods. Absence or presence (0 = absence, 1 = presence) of (a) sexual abuse (whether involving physical contact or not and including sexual exploitation); (b) psychological abuse (e.g., threats, denigration, and emotional rejection); and (c) physical abuse (e.g., excessive physical punishment causing bodily injury) were considered.

*Behavior problems.* The presence of various nonsexual behavior problems was recorded to make it possible to examine their occurrence as well as possible association with the development of SBP. The absence or presence (0 = absence, 1 = presence) of the following behaviors were examined for each developmental period: (a) persistent aggression (e.g., verbal and physical aggression such as threatening or hitting others); (b) delinquency (e.g., vandalism and theft); (c) running away; and (d) self-harm and/or suicidal thoughts.

*Occurrence of SBP.* SBP was coded based on any instance reported by a CPS professional for a child younger than 18 years of age. In order to minimize issues related to false negatives (i.e., a young person having been incorrectly determined as not displaying SBP), the entire CPS file was examined. This examination made it possible to ensure that coding for SBP occurrences was not limited to instances where the referral to CPS was specifically related to SBP-related events. For example, a CPS professional investigating a case of parental neglect might have noted that one of the victims exhibited SBP. In Quebec, CPS distinguishes between two types of SBP: (a) inappropriate sexual behaviors and (b) sexual violence, both of which are considered in the current study. Inappropriate sexual behavior refers to behaviors of a sexual nature that can jeopardize a child’s safety and/or development (e.g., involvement in the production/distribution of child pornography, using child pornography, gross indecency, and inciting someone to participate in a sexual act). Sexual violence refers to coercive and/or aggressive sexual contact with a victim.

To examine the development of SBP, data were collected regarding the age of onset as well as the continuity or discontinuity of these behaviors during childhood and adolescence. Information about age at the time of the first occurrence of SBP was used to determine if the young person began to exhibit SBP during childhood or in adolescence (0 = adolescence-onset; 1 = childhood-onset). Two groups were identified based on age of onset: (a) a group of young people whose SBP began in adolescence (*n* = 182), and (b) a group of young people whose SBP began during childhood (*n* = 158). Based on information in the case files, the childhood-onset group had a mean age of onset of 9.3 (*SD* = 1.8; Range: 4–11) while the mean age of onset for the adolescence-onset group was 13.8 (*SD* = 1.4; Range: 12–17). Only 1.2% of the childhood-onset group showed SBP beginning before the age of six. Statistical analyses were conducted on the sample and the statistical models were designed to discriminate between childhood-onset and adolescence-onset. Continuity of SBP was determined based on an occurrence of SBP in both childhood and adolescence (0 = discontinuity; 1 = continuity). This made it possible to identify two groups: (a) one group in which SBP was limited to childhood (*n* = 81), and (b) one group in which SBP began during childhood and persisted into adolescence (*n* = 77). Because the study focuses on the continuity and discontinuity of childhood-onset SBP, those whose SBP was limited to adolescence were not included in the second step of classification.

## Analytic Strategy

A series of logistic regressions were conducted to identify possible developmental covariates associated with childhood-onset SBP and its continuity into adolescence. For exploratory purposes, a series of logistic regression models were conducted to consider the developmental covariates at each of the developmental stages examined. Separate regression models were conducted for the periods of infancy, early childhood, and late childhood. The standard approach of combining all covariates into a single regression model was problematic in the current study as the developmental covariates examined are not independent, which could create several statistical issues (e.g., multicollinearity). A stepwise statistical approach (e.g., Hosmer & Lemeshow, 2000; Tabachnick & Fidell, 2013) was therefore followed to complement the standard logistic regression analyses, making it possible to determine the most statistically significant covariates for SBP during simultaneous analysis of all covariates. While taking a purely statistical approach in creating a prediction model of a social phenomenon is never a good idea, the goal of the current study was not to create either a prediction model or a prediction instrument. The stepwise method was used in the current study as an exploratory method that makes it possible to look at a limited set of developmental factors to examine whether some of these factors are more strongly related to the onset and continuity of SBP. While there are well-known limitations to the stepwise approach (e.g., Henderson & Denison, 1989), measures were taken to minimize their impact^2^.

For the stepwise regression analyses, a hierarchical approach was followed, with a different set of covariates added to the model sequentially according to their chronological order of appearance in the lives of those in the sample. For each regression model, priority of entry was given to control variables that were forced into the regression model. Following forced entry of the control variables, a hierarchical approach was used, which made it possible to prioritize earlier developmental covariates. More exactly, each set of developmental covariates corresponded to an indicator characterizing covariates at a specific developmental period (i.e., infancy, early childhood, and late childhood). A new set of developmental covariates was added to the previous set of covariates at each subsequent step of the analysis, mirroring the experiences and development of the child over time. For each set of developmental covariates, a stepwise forward selection method was used to include covariates based on their statistical significance to the outcome. This method made it possible to identify the covariates that are significant in terms of predicting the outcome (Hosmer & Lemeshow, 2000). Odds ratio (OR) was used to examine the association between covariates and outcome. The effect of a developmental covariate was interpreted based on whether it increased (OR > 1) or decreased (OR < 1) the likelihood of the outcome (Tabachnick & Fidell, 2013).

Because the study is based on a subsample of the population during the study period, unweighted and weighted regression models were examined. The unweighted coefficients reflect the data sample while the weighted coefficients include a weight to reflect the actual proportions of childhood only SBP and SBP that began in childhood and persisted into adolescence in the targeted population (*n* = 1933). This adjustment was made to address a methodological limitation observed in prior studies that are based on convenient samples without adjusting for the effect of the regression coefficients. Model fit and adequacy were assessed by different methods. For goodness of fit, the Hosmer–Lemeshow test was used and was not significant (i.e., *p* > .05), suggesting the model fits the data well (Hosmer & Lemeshow, 2000). To assess the adequacy of the model in accurately predicting the outcome, classification tables and pseudo R^2^ were calculated to verify improvement in the model likelihood following inclusion of developmental covariates. Assumptions (e.g., problems related to data sparseness or multicollinearity) were tested prior to the analyses to confirm statistical analysis decisions and improve statistical power (Tabachnick & Fidell, 2013). To avoid problems related to deficits in the data, only independent variables with a cell count of at least five observations in contingency tables were added in the multivariate analyses. This led to the exclusion of the variables of sexual and psychological abuse in infancy; inadequate sexual boundaries between parent and child in infancy and early and late childhood; and running away in late childhood. Presence of multicollinearity among covariates was examined based on variance inflation factors (VIF). No evidence of multicollinearity problems was detected, given that VIF values were higher than one and under 10 (Mansfield & Helms, 1982). Finally, multivariate outliers were screened and five were identified. We then replicated the logistic regression models after removing outlying cases. Because no significant difference was noted with regard to model fit, these cases were retained (Hosmer & Lemeshow, 2000)^3^.

## Results

### Bivariate Analyses of Developmental Correlates of SBP

Using Spearman’s rho coefficients, correlations between covariates and developmental aspects of SBP (i.e., childhood-onset SBP and continuity of childhood SBP into adolescence) were explored. These preliminary analyses made it possible to identify potential intervening and indirect effects between developmental factors and SBP. The results are presented in Table 2.

**Table 2.** Spearman Correlations of Developmental Factors for Childhood-Onset and Continuity of sexual behavior problems.

Variables	Childhood-Onset			Continuity		
	Infancy	Early Childhood	Late Childhood	Infancy	Early Childhood	Late Childhood
Family environment						
History of placement	−.04	.02	.16**	.14	.13	.21**
Parental drug/alcohol abuse	−.00	.08	.08	−.03	.06	.02
Parental long-term absence	−.02	−.08	−.07	.07	.12	.12
Parental antisocial associations	.03	.09	.12*	.01	.09	.15
Parental impulsivity, instability	.08	.10	.17**	−.02	.07	.15
Inadequate sexual boundaries	.08	.13*	.21**	.12	.04	.12
Parental neglect	−.01	.11*	.14**	.06	.03	.26**
Exposure to intimate partner violence	.14**	.18**	.15**	.01	.07	−.06
History of victimization						
Sexual abuse	–	−.02	.06	–	.04	.13
Psychological abuse	–	.11*	.14**	–	.20*	.08
Physical abuse	–	.08	.13*	.05	.12	.04
Behavior problems						
Persistent aggression	–	–	.39**	–	–	.19*
Delinquency	–	–	.22**	–	–	.22**
Self-harm/Suicidal thoughts	–	–	.14*	–	–	.10
Running away	–	–	.10	–	–	.18*
Control variables	Childhood-Onset			Continuity		
Sex	.21**			.03		
Ethnicity	−.11			.08		
Early age at 1^st^ CPS referral	.07			.27**		
Siblings	.01			.06		
Parental criminal record	.06			.18*		
Number of CPS referrals	.07			.27**		

*Note.* Sample size varies between 292 and 340 due to missing data.

*p < .05; **p < .01.

For control variables, only the child’s sex was significantly correlated with SBP, suggesting that boys are more likely to show a childhood-onset of SBP. It was noted that the number of relevant developmental correlates increased from infancy to late childhood, revealing an accumulation of factors over time. In late childhood, most of these factors were significantly associated with a childhood-onset of SBP. One factor, being exposed to intimate partner violence, was significantly associated with childhood-onset SBP following exposure in any of the three periods. Exposure to inadequate parental sexual boundaries, experiencing neglect, and psychological abuse were somewhat significant across two periods—early and late childhood. Sexual abuse, as opposed to physical and psychological abuse, was the only type of victimization not significantly correlated to the onset of SBP in any period. Finally, most of the nonsexual behavior problems in late childhood (i.e., persistent aggression, delinquency, and self-harm/suicidal thoughts) were statistically associated with a childhood-onset of SBP.

Significant statistical associations with SBP that continued into adolescence were noted for three control variables—early age at first referral to CPS, parental criminal record, and total number of CPS referrals. Experiencing psychological abuse was the only ACE significantly correlated with continuity of SBP. With regard to family environment, history of placement and parental neglect during late childhood were significant correlates. Finally, SBP that continued into adolescence was significantly associated with three nonsexual behavior problems—persistent aggression, delinquency, and running away—in late childhood.

### Multivariate Analyses of the Developmental Covariates

A series of regressions were conducted to examine the timing of developmental covariates, first for age of onset of SBP and then for continuity of SBP. Each regression models was examined using unweighted and weighted regression coefficients, reported below.

*Developmental covariates of childhood-onset of SBP.* Standard logistic regression models were conducted to examine the timing of the developmental covariates of age of onset of SBP for the three developmental periods examined in the current study (Table 3). For the period of infancy, after adjusting for control variables, none of the developmental covariates was statistically significant in the unweighted regression model although some trends were noticeable. These trends were statistically significant in the weighted model for two covariates—parental association with antisocial peers (OR = 2.14) and parental impulsivity and instability (OR = 2.13)—and were associated with a childhood-onset of SBP. For the weighted model, childhood-onset of SBP was associated with lower odds of a history of placement (OR = .35) suggesting that placement was significantly more common among youth with adolescence-onset SBP. For both the unweighted and weighted models, Nagelgerke’s pseudo R^2^ was very low (.01 and .07, respectively).

**Table 3.** Logistic Regression Analyses of the Developmental Covariates for Childhood-Onset of Sexual Behavior Problems.

	Unweighted Models			Weighted Models		
Covariates	Model 1Infancy	Model 2Early Childhood	Model 3Late Childhood	Model 1Infancy	Model 2Early Childhood	Model 3Late Childhood
	OR (95% C.I.)	OR (95% C.I.)	OR (95% C.I.)	OR (95% C.I.)	OR (95% C.I.)	OR (95% C.I.)
Infancy						
Parental drug/alcohol abuse	.41 (.13–1.27)	-	-	.64 (.39–1.05)	-	-
History of placement	.54 (.18–1.65)	-	-	.35 (.20–.62)**	-	-
Parental long-term absence	.87 (.34–2.21)	-	-	.86 (.57–1.32)	-	-
Parental antisocial associations	2.39 (.61–9.42)	-	-	2.14 (1.17–3.92)*	-	-
Parental impulsivity, instability	1.70 (.44–6.60)	-	-	2.13 (1.13–4.02)*	-	-
Early childhood						
Parental drug/alcohol abuse	*-*	.71 (.28–1.83)	-	-	1.09 (.71–1.66)	-
History of placement	*-*	.76 (.33–1.75)	-	-	.65 (.43–.98)*	-
Parental long-term absence	*-*	.54 (.26–1.14)	-	-	.42 (.29–.60)***	-
Parental antisocial associations	*-*	1.77 (.56–5.55)	-	-	2.01 (1.18–3.43)*	-
Parental impulsivity, instability	*-*	.73 (.25–2.17)	-	-	.92 (.53–1.61)	-
Exposure to intimate partner violence	*-*	.68 (.05–9.48)	-	-	1.32 (.38–4.60)	-
Parental neglect	*-*	1.80 (.61–5.34)	-	-	3.53 (2.08–5.98)***	-
Psychological abuse	*-*	4.34 (.40–47.48)	-	-	2.80 (.95–8.23)	-
Physical abuse	*-*	1.15 (.35–3.75)	-	-	1.46 (.80–2.66)	-
Late childhood						
Parental drug/alcohol abuse	*-*	*-*	.98 (.40–2.38)	*-*	*-*	1.16 (.77–1.75)*
History of placement	*-*	*-*	.91 (.43–1.91)	*-*	*-*	.93 (.65–1.32)
Parental long-term absence	*-*	*-*	.48 (.24–.94)*	*-*	*-*	.38 (.27–.53)***
Parental antisocial associations	*-*	*-*	1.48 (.59–3.69)	*-*	*-*	1.56 (1.00–2.41)*
Parental impulsivity, instability	*-*	*-*	.74 (.35–1.56)	*-*	*-*	.60 (.41–.86)**
Parental neglect	*-*	*-*	.96 (.45–2.04)	*-*	*-*	.45 (.30–.65)***
Exposure to intimate partner violence	*-*	*-*	.72 (.18–2.83)	*-*	*-*	1.14 (.59–2.20)
Sexual abuse	*-*	*-*	1.81 (.78–4.16)	*-*	*-*	1.29 (.86–1.93)
Psychological abuse	*-*	*-*	2.74 (.75–9.99)	*-*	*-*	2.62 (1.39–4.95)**
Physical abuse	*-*	*-*	1.16 (.50–2.72)	*-*	*-*	1.38 (.93–2.04)
Delinquency	*-*	*-*	3.18 (1.18–8.55)*	*-*	*-*	2.63 (1.64–4.22)***
Self-harm/Suicidal thoughts	*-*	*-*	1.07 (.43–2.67)	*-*	*-*	.99 (.65–1.50)
Persistent aggression	*-*	*-*	5.45 (2.67–11.12)***	*-*	*-*	5.54 (3.94–7.77)***
Control variables						
Sex	2.79 (1.61–4.85)***	2.75 (1.55–4.87)**	2.27 (1.22–4.22)**	2.55 (1.98–3.29)***	2.49 (1.91–3.27)***	2.60 (1.94–3.47)***
Ethnicity	.38 (.11–1.26)	.35 (.10–1.21)	.34 (.09–1.34)	.23 (.11–.44)***	.17 (.08–.36)***	.24 (.12–.50)***
Early age at 1^st^ CPS referral	0.96 (.51–1.79)	.87 (.41–1.84)	.77 (.37–1.59)	.60 (.44–.80)**	.37 (.25–.54)***	.55 (.39–.77)***
Siblings	.85 (.70–1.02)	.84 (.69–1.01)	.80 (.66–.99)*	.84 (.77–.91)***	.80 (.73–.88)***	.82 (.75–.90)***
Parental criminal record	1.19 (.66–2.17)	1.04 (.57–1.89)	.79 (.39–1.60)	.83 (.63–1.09)	.71 (.54–.95)*	.51 (.37–.72)***
Number of CPS referrals	1.07 (1.01–1.12)*	1.05 (.99–1.11)	1.02 (.95–1.09)	1.07 (1.04–1.09)***	1.04 (1.02–1.07)**	1.05 (1.02–1.08)**
Constant	.41**	.48*	.40*	.36***	.47***	.28***
Model fit						
Nagelkerke pseudo R^2^	.01	.09	.21	.07	.11	.20
Model coefficients X^2^ (df)	28.13 (11)**	36.55 (15)**	81.51 (19)***	144.31 (11)***	222.76 (15)***	389.28 (19)***

*Note.* Sample size for these analyses is 285. CI = confidence interval; OR = odds ratio*.*

*p < .05; **p < .01; ***p < .001

For the early childhood period, after adjusting for control variables, none of the developmental covariates of the unweighted model were statistically significant, although some trends were noticeable. For the weighted models, however, four developmental covariates were statistically significant. Childhood-onset of SBP was associated with higher odds of parental association with antisocial peers (OR = 2.01) and parental neglect (OR = 3.53). The models also showed that youth with childhood-onset SBP were less likely to have had a history of placement (OR = .65) and had lower odds of having had to deal with the long-term absence of a parent (OR = .42). That said, the Nagelgerke’s pseudo R^2^ for the unweighted and weighted models were relatively low (.09 and .11, respectively).

Finally, for the late childhood period, statistically significant developmental covariates were identified for both the unweighted and weighted models. Both models showed that childhood-onset SBP was statistically associated with higher odds of signs of persistent aggression (unweighted OR = 5.45; weighted OR = 5.54) and childhood delinquency (unweighted OR = 3.18; weighted OR = 2.63) but lower odds of long-term parental absence (unweighted OR = .48; weighted OR = .38). The weighted model identified other statistically significant covariates, showing that childhood-onset SBP was more likely to occur in a context of psychological abuse (weighted OR = 2.62) and parental drug/alcohol abuse (weighted OR = 1.16) or antisocial peer association (weighted OR = 1.56). The model also showed that, compared to those with adolescence-onset SBP, youth with childhood-onset SBP were less likely to have been subject to long-term parental absence (weighted OR = .38), parental impulsivity and instability (weighted OR = .60), or parental neglect (weighted OR = .45). The Nagelgerke’s pseudo R^2^ for the unweighted and weighted models was higher for the late childhood period (.21 and .20, respectively). For control variables, the child’s sex remained statistically significant across all regression models tested, suggesting that those with childhood-onset SBP were more likely to be boys.

The same set of factors were re-analyzed using a hierarchical stepwise approach to examine the developmental covariates of childhood-onset SBP (see Supplementary Table 4; these analyses should be understood as exploratory and complementary to those previously presented). A single logistic regression was conducted and developmental covariates were entered in stages, starting with infancy, followed by early childhood and then late childhood covariates. Throughout this process, regression models were adjusted for all statistical controls (e.g., child’s sex, ethnicity, and presence of siblings). In the first model, only the control variables were significant. Although the model was statistically significant, the Nagelkerke’s pseudo R^2^ was very low (.06). Entering early childhood covariates significantly improved the fit of the logistic regression model although the Nagelkerke pseudo R^2^ remained relatively low (.08). Only one developmental covariate—being exposed to psychological abuse (unweighted OR = 3.60)—was related to childhood-onset SBP. Entering the developmental covariates for the late childhood period significantly improved the fit of the model, with the Nagelgerke’s pseudo R^2^ improving to .20. Youth with childhood-onset SBP had significantly higher odds of having experienced psychological abuse (unweighted OR = 2.21) as well as of showing signs of persistent aggression (unweighted OR = 2.62) and childhood delinquency (unweighted OR = 5.43). This analysis seems to suggest that the developmental covariates for late childhood were more strongly associated with childhood-onset SBP, indicating that the set of developmental covariates included in the final model improved the model’s ability to discriminate between childhood-onset and adolescence-onset SBP. Note that the regression coefficients between the unweighted and weighted analyses were relatively stable, the statistically significant covariates were identical, and the ability to discriminate between childhood and adolescence-onset was very similar if not almost identical. Although the goal of the study was not to create a prediction model for SBP, the percentage of overall correct classification is often used to determine the logistic model’s ability to discriminate between childhood and adolescence-onset SBP based on the covariates used. This final unweighted regression model was significant and showed an overall percentage of correct classification of 73.0% (i.e., 83.7% of the adolescence-onset group and 61.6% of the childhood-onset group), which is well above chance.

*Developmental covariates of the continuity of SBP.* Another series of standard logistic regression analysis were conducted to examine the timing of the developmental covariates for continuity of SBP into adolescence (Table 4). Separate regression models were conducted for the three developmental periods examined. For the infancy period, after adjusting for control variables, only one developmental covariate—parental drug and/or alcohol abuse (OR = .08)—was statistically significant for the unweighted regression model. Continuity of SBP was associated with lower odds of parental drug and/or alcohol abuse, suggesting that this abuse was significantly more common for those whose SBP did not continue into adolescence. For the weighted models, two developmental covariates were statistically significant: parental drug and/or alcohol abuse (OR = .05) and a history of out-of-home placement (OR = 5.84). Continuity of SBP into adolescence was associated with higher odds of out-of-home placement but lower odds of parental drug and/or alcohol abuse. For both the unweighted and weighted models, Nagelgerke’s pseudo R^2^ was low (.14 for both models).

**Table 4.** Logistic Regression Analyses of the Developmental Covariates for the Continuity of Sexual Behavior Problems.

	Unweighted Models			Weighted Models		
Covariates	Model 1Infancy	Model 2Early Childhood	Model 3Late Childhood	Model 1Infancy	Model 2Early Childhood	Model 3Late Childhood
	OR (95% C.I.)	OR (95% C.I.)	OR (95% C.I.)	OR (95% C.I.)	OR (95% C.I.)	OR (95% C.I.)
Infancy						
Parental drug/alcohol abuse	.08 (.01–.66)*	-	-	.05 (.01–.40)**	-	-
History of placement	4.16 (.74–23.35)	-	-	5.84 (1.33–25.64)*	-	-
Parental long-term absence	.89 (.25–3.22)	-	-	.92 (.30–2.84)	-	-
Parental antisocial associations	1.73 (.23–13.14)	-	-	2.74 (.40–18.85)	-	-
Parental impulsivity, instability	.69 (.14–3.48)	-	-	.66 (.15–2.81)	-	-
Early childhood						
Parental drug/alcohol abuse	*-*	.10 (.01–.66)*	-	-	.03 (.01–.21)***	-
History of placement	*-*	1.03 (.28–3.76)	-	-	1.20 (.37–3.90)	-
Parental long-term absence	*-*	1.32 (.43–4.09)	-	-	1.25 (.43–3.59)	-
Parental antisocial associations	*-*	1.36 (.27–6.92)	-	-	1.78 (.44–7.21)	-
Parental impulsivity, instability	*-*	.54 (.14–2.12)	-	-	.46 (.13–1.62)	-
Exposure to intimate partner violence	*-*	.98 (.09–10.78)	-	-	2.22 (.20–24.40)	-
Parental neglect	*-*	.63 (.17–2.33)	-	-	.64 (.19–2.09)	-
Psychological abuse	*-*	.83 (.09–7.76)	-	-	.30 (.03–2.58)	-
Physical abuse	*-*	1.25 (.29–5.45)	-	-	1.16 (.32–4.18)	-
Late childhood						
Parental drug/alcohol abuse	*-*	*-*	.27 (.09–.81)*	*-*	*-*	.24 (.09–.65)**
History of placement	*-*	*-*	1.42 (.60–3.40)	*-*	*-*	1.28 (.62–2.64)
Parental long-term absence	*-*	*-*	1.57 (.66–3.77)	*-*	*-*	2.38 (1.10–5.18)*
Parental antisocial associations	*-*	*-*	2.32 (.74–7.32)	*-*	*-*	3.06 (1.08–8.69)*
Parental impulsivity, instability	*-*	*-*	1.96 (.77–4.99)	*-*	*-*	2.13 (.95–4.79)
Parental neglect	*-*	*-*	3.77 (1.41–10.10)***	*-*	*-*	4.10 (1.71–9.81)**
Exposure to intimate partner violence	*-*	*-*	.15 (.04–.65)*	*-*	*-*	.12 (.04–.41)**
Sexual abuse	*-*	*-*	2.88 (1.05–7.91)*	*-*	*-*	3.84 (1.59–9.29)**
Psychological abuse	*-*	*-*	2.07 (.58–7.38)	*-*	*-*	1.96 (.73–5.28)
Physical abuse	*-*	*-*	1.02 (.38–2.78)	*-*	*-*	.83 (.36–1.92)
Delinquency	*-*	*-*	1.19 (.46–3.11)	*-*	*-*	1.28 (.55–2.98)
Self-harm/Suicidal thoughts	*-*	*-*	.97 (.36–2.63)	*-*	*-*	1.11 (.47–2.63)
Persistent aggression	*-*	*-*	.77 (.32–1.83)	*-*	*-*	.71 (.32–1.58)
Control variables						
Sex	1.42 (.64–3.16)	1.79 (.76–4.24)	1.30 (.50–3.36)	1.50 (.73–3.05)	2.01 (.88–4.60)	1.40 (.59–3.30)
Ethnicity	2.14 (.27–17.21)	2.11 (.28–16.14)	3.27 (.35–30.69)	2.83 (.57–14.08)	2.52 (.50–12.73)	4.35 (.66–28.46)
Early age at 1^st^ CPS referral	7.09 (2.78–18.08)***	19.55 (4.51–84.74)***	4.97 (1.90–12.98)**	7.37 (3.64–14.90)***	49.20 (12.58–192.44)***	4.95 (2.27–10.81)***
Siblings	.96 (.72–1.28)	.99 (.76–1.30)	0.89 (.67–1.19)	.98 (.76–1.26)	1.03 (.80–1.32)	.88 (.68–1.13)
Parental criminal record	1.99 (.93–4.27)	2.28 (1.04–5.00)*	2.43 (1.05–5.62)*	1.99 (1.07–3.71)*	2.58 (1.31–5.09)**	2.87 (1.43–5.76)**
Number of CPS referrals	1.00 (.95–1.06)	1.01 (.95–1.07)	0.93 (.86–1.02)	1.01 (.96–1.06)	1.01 (.96–1.07)	.95 (.88–1.02)
Constant	.24**	.17**	0.20**	.06***	.04***	.04***
Model fit						
Nagelkerke Pseudo R^2^	.14	.15	.21	.14	.16	.21
Model coefficients X^2^ (df)	36.37 (11)***	38.71 (15)***	56.87 (20)***	57.93 (11)***	69.66 (15)***	90.72 (19)***

*Note*. Sample size for these analyses is 138. CI = confidence interval; OR = odds ratio.

*p < .05; **p < .01; ***p < .001

For the period of early childhood, after adjusting for control variables, parental drug and/or alcohol abuse was the only statistically significant developmental covariate for the unweighted regression model (OR = .10) and the weighted regression model (OR = .03). Both the unweighted and weighted models showed that continuity of SBP into adolescence was associated with lower odds of parental drug and/or alcohol abuse in early childhood, suggesting that this abuse was significantly more common for children whose SBP was limited to childhood. The Nagelgerke’s pseudo R^2^ for the unweighted and weighted models remained relatively low for the early childhood period (.15 and .16, respectively).

Finally, for the late childhood period, statistically significant developmental covariates were identified for both the unweighted and weighted models. Both models showed that the continuity of SBP into adolescence was statistically associated with higher odds of parental neglect (unweighted OR = 3.77; weighted OR = 4.10) and experiencing sexual abuse (unweighted OR = 2.88; weighted OR = 3.84), but lower odds of parental drug and/or alcohol abuse (unweighted OR = .27; weighted OR = .24) and exposure to intimate partner violence (unweighted OR = .15; weighted OR = .12). The weighted model identified other statistically significant covariates, showing that continuity of SBP into adolescence was associated with higher odds of having had to deal with long-term parental absence (weighted OR = 2.38) and parental association with antisocial peers (weighted OR = 3.06). The Nagelgerke’s pseudo R^2^ for the unweighted and weighted models was higher for the late childhood period (.21 for both unweighted and weighted models). Note that among the control variables examined, early age at first CPS referral remained statistically significant across all regression models tested, suggesting that first referral to CPS for youth with SBP that continued from childhood into adolescence was more likely to have occurred before 6 years of age.

The same set of factors were re-analyzed using a hierarchical stepwise approach to examine the developmental covariates of the continuity of SBP (see Supplementary Table 6). In the first model, parental drug and/or alcohol abuse (unweighted OR = .16) was the only statistically significant developmental covariate other than control variables. Although the model was statistically significant, the Nagelkerke’s pseudo R^2^ for this initial model was low (.12). Developmental covariates for the early childhood period were entered but only the control variables were statistically significant and the Nagelkerke’s pseudo R^2^ for this initial model remained low (.12) and statistically significant. Entering the developmental covariates for the late childhood period significantly improved the fit of the model, with the Nagelgerke’s pseudo R^2^ improving to .17. Youth with persistent SBP had significantly higher odds of having experienced parental neglect (unweighted OR = 2.86) but lower odds of having been exposed to intimate partner violence (unweighted OR = .32). Note that among the control variables examined, early age at first CPS referral remained statistically significant across all regression models tested, suggesting that youth with SBP that continued from childhood into adolescence were more likely to have been first referred to CPS before 6 years of age.

Overall, the results appear to indicate that the set of developmental covariates related to the late childhood period improved the final model’s ability to discriminate between childhood-limited SBP and SBP that continued into adolescence. The final unweighted regression model was significant and overall percentage of correct classification was 71.7% (i.e., 74.2% of the childhood-limited group and 69.4% of the continuity group), which is well above chance. The unweighted and weighted regression models were relatively similar but there were three significant differences that demonstrated the importance of using the weighted approach. First, a history of out-of-home placement in late childhood (weighted OR = 2.74) emerged as a significant covariate of continuation—youth with SBP that continued into adolescence had significantly higher odds of having experienced an out-of-home placement in late childhood. Second, parental drug and/or alcohol abuse in early childhood (weighted OR = .17) became a statistically significant covariate for continuation in that youth with at least one parent who demonstrated such problems were least likely to show childhood SBP that continued into adolescence. Third, among control variables, parental criminal record was a significant covariate of persistence of SBP into adolescence (weighted OR = 2.68).

## Discussion

This study investigated the developmental antecedents of childhood SBP as well as antecedents associated with the persistence of SBP into adolescence. Based on retrospective-longitudinal data for a sample of youth referred to CPS, the present findings show that children whose SBP started in childhood and persisted into adolescence have often experienced various adversities in the family environment and tend to display nonsexual behavior problems. Findings also make it possible to provide some details about the processes through which such adversity affects the development of SBP in childhood.

### The Developmental Context of Childhood SBP

Attachment theorists have frequently argued about the role and importance of ACE in the etiology of sexual offending (e.g., Marshall & Barbaree, 1990; Smallbone, 2006). The findings in the current study provide further empirical evidence that ACE are not only prevalent in the lives of youth with SBP who have been referred to CPS (e.g., Lussier et al., 2019) but are also statistically significantly associated with childhood SBP and its persistence into adolescence. The study findings provide more detail about the developmental context surrounding childhood SBP that persists into adolescence and reveal that persistence of SBP is characterized by various developmental factors (see also Vizard, Hickey, French, & McCrory, 2007; Vizard, Hickey, McCrory 2007) as results show that persistent SBP appear to be strongly related to ACE that affect the child directly (e.g., neglect in late childhood), rather than to problems that characterize the child’s family environment (parental drug/alcohol abuse during infancy, exposure to intimate partner violence in late childhood) and to which children with persistent SBP tend to have been less exposed. Findings also suggest that children with persistent SBP are more prone to have entered the CPS system at a very early age, so it is possible that this finding is related to the specific forms of intervention (out-of-home placement) or monitoring prescribed by CPS under certain circumstances (e.g., seriousness of the situation; child’s age and personal characteristics); interventions that may lower exposure to difficulties related to the family environment. It is also possible that children in various family situations, such as those captured by the covariates for childhood SBP, may have been removed from home and put into a placement that inadvertently provided a context conducive to the persistence of SBP (e.g., presence of vulnerable children in the new environment). Placement in an alternative living environment, whether as a response to children’s behavior problems or to difficulties in the family environment, thus appears to be both an important component in the developmental context of persistent SBP and a potential associated factor.

Based on the study findings, a number of observations can be made about the development of childhood SBP and its continuity into adolescence. First, after statistically controlling for exposure to ACE, findings showed that young boys are more prone to a pattern of childhood SBP that persists into adolescence than young girls. Second, findings provide additional evidence that children with childhood SBP tend to show nonsexual externalized behavior problems, particularly persistent aggression and delinquency, perhaps because the developmental factors responsible for these externalizing behavior problems are similar to those related to the onset and persistence of SBP. Third, developmental covariates in the period marking the onset of SBP appear to be important in understanding childhood SBP and its persistence into adolescence. For the sample considered here, childhood SBP occurred almost exclusively during late childhood; developmental covariates were more strongly associated statistically with both childhood-onset and continuity into adolescence during this period than in prior developmental periods. In other words, it appears that childhood-onset and continuity of SBP might be better explained at this early age by proximal rather than more distal factors (i.e., by factors that affect the child directly rather than being related to family environment). Note that the models tested do not prove that the ACE examined are causal, only that they were more likely to be part of children’s developmental context. Fourth, the findings show that the developmental covariates for childhood SBP and for continuity of SBP into adolescence are relatively distinct. For example, while externalizing behavior problems were statistically associated with childhood-onset SBP, they were not associated with continuity of SBP into adolescence. This highlights the importance of approaching childhood SBP and continuity of SBP into adolescence as distinct phenomena.

The findings have important clinical implications for intervention with children with SBP who are referred to CPS, demonstrating the need to consider a broad set of developmental needs related to family environment, history of victimization, and behavior during assessment and intervention–clinical assessment of children with SBP needs to go well beyond the issue of sexual victimization experiences (Elkovitch et al., 2009; Lussier et al., 2019; Merrick et al., 2008). Our findings suggest that research in this area could benefit from closer attention to the issue of complex trauma, sometimes called complex trauma exposure, which involves multiple, chronic, and prolonged ACE (e.g., Cook et al., 2005). It has been suggested that complex trauma may have an effect on both the self-regulation of emotions, impulses, and behavior and the development of social competence (e.g., Kinniburgh et al., 2017; Wamser-Nanney & Vanderberg, 2013). Children repeatedly exposed to ACE may continue to develop new symptoms as they are faced with new stressors, additional developmental challenges, and different social contexts (e.g., entry into elementary school). Conversely, findings suggest that the clinical assessment of children exposed to ACE or showing signs of complex trauma should include examination of sexual development even in contexts where there is little reason to believe that the child has been sexually abused. Current clinical assessment tools, such as the Child Behavior Checklist (CBCL; Achenbach & Ruffle, 2000), do not capture important clinical information about a child’s sexual development as related to SBP and its development.

Prior research has stressed the role and importance of experiences of sexual victimization in the lives of children with SBP (Friedrich et al., 1991, 1992) and has suggested that it is a key developmental antecedent for sexual offending in adolescence (e.g., Burton et al., 1997; Hawkes, 2011). The current study does not challenge these earlier reports of the role and importance of sexual victimization experiences, particularly as our sample did not include children with no evidence of SBP. However, our thorough review of CPS files suggests that future research should more closely examine the familial environment of children with SBP. Prior research has shown that parents of children with SBP tend to show poor parental control (e.g., limited supervision), a lack that is particularly problematic in a context where the familial environment is chaotic and disorganized (Pithers et al., 1998), and have stressed the possible cumulative effect of adverse early life experiences, suggesting that the developmental context surrounding the occurrence of SBP among children is characterized by complex trauma exposure that is not the result of sexual victimization alone (e.g., Szanto et al., 2012; Tarren-Sweeney, 2008). The co-occurrence of different ACE in the lives of children may increase their vulnerability to developing SBP. For example, parental neglect may inadvertently create a context that favors sexual victimization by a babysitter, a family friend, or a family member, which in turn may contribute to the onset of SBP. Disentangling the role and importance of multiple ACE is a difficult task that cannot be resolved in a single study. Based on the finding that not all children who exhibit SBP have experienced sexual victimization, it appears important to take a more in-depth look at the association between SBP and nonsexual trauma experiences and factors (e.g., Elkovitch et al., 2009).

### Limitations and Future Directions

Although the developmental perspective of the current study is important, some methodological limitations should be discussed. First, analyses were based on official data retrieved from CPS files in the province of Quebec, Canada. Findings thus deal only with the influence of reported forms of abuse and maltreatment and may reflect Quebec’s specific sociolegal context, meaning that they are not completely generalizable. Moreover, the vast majority of the sample was French-speaking and White, which limited examination of potentially relevant and important cultural factors. Other empirical studies have pointed to differences in adverse experiences in childhood across ethnic groups and their relation to future delinquency (e.g., DeLisi et al., 2017). Further investigations should be conducted in different sociolegal contexts to assess the representativeness of our findings. Nonetheless, one important advantage of our data collection method is its longitudinal nature: CPS professionals recorded information about the youth with whom they were involved from birth to age 17. This methodology made it possible to conduct a longitudinal investigation of the developmental covariates of SBP across childhood and adolescence. This collection method, however, limited the sample to youth who had been referred to CPS and the assessment of SBP thus relied on the judgment of a CPS professional rather than a reliable and valid instrument such as the Child Sexual Behavior Inventory (Friedrich et al., 1992). The results thus reflect CPS practices in Quebec. Another limitation regards the size of the subgroups created on the basis of age of onset and continuity/discontinuity of SBP. A larger number of youth whose SPB was limited to childhood were part of our sample. This increased the statistical power of the analyses and was useful in providing a sample sufficient to allow us to conduct a detailed investigation of the developmental covariates associated with childhood SPB and its persistence into adolescence, but to counterbalance its effect, unweighted and weighted regressions models were examined and the findings reported. Finally, this is an exploratory study of the role and importance of ACE for childhood SBP and its persistence into adolescence. Our findings do not show that the developmental covariates examined here have a causal effect on childhood SBP and its continuity into adolescence. Future research should go beyond examination of the presence and timing of ACE and their statistical significance to include more detailed information about the context, nature, and frequency of such experiences, such as examining the type and frequency of out-of-home placements and their relation to the onset and continuity of SBP.

## Conclusion

This study provides additional information about the adverse early life experiences suffered by youth who exhibit SBP and their role in the development of these behaviors across childhood and adolescence. In contrast to most past studies, a longitudinal design was used to allow a thorough examination of the influence of difficulties in early life on childhood SBP and its persistence into adolescence among a sample of youth referred to CPS. Findings provide additional information about the developmental context for SBP, such as recurrent experiences of adversity and co-occurring nonsexual behavior problems across different age periods in childhood. The study findings provide further empirical evidence that can be used to create a developmental theory–based model of SBP. While such a developmental theory of SBP is not yet available, it is clear that ACE are a key component of any model that attempts to explain childhood SBP and its continuity into adolescence. Future research should focus on obtaining a better understanding of the association between ACE and SBP, as well as of the mechanisms responsible for such an association. Intervention and prevention methods must be developmentally sensitive and appropriate, taking into account factors that are influential during specific age periods. Findings here underline the value of timely trauma-informed care for children and youths who exhibit SBP (Daignault & Brend, in press; Levenson, 2014). The Substance Abuse and Mental Health Services Administration (SAHMSA, 2014) defines trauma-informed care as intervention approaches that realize the importance and impact of trauma, recognize its signs, respond accordingly, and take advantage of all available means to resists re-traumatization. Our results suggest that trauma-informed care may reduce the likelihood that childhood SBP will persist into adolescence for children referred to CPS. Lastly, both trauma and developmentally informed research based on early life experiences is clearly needed to better understand both the factors involved and effective interventions for this population.

## Supplemental Material

sj-pdf-1-sax-10.1177_10790632211047184 – Supplemental Material for A Longitudinal Examination of Developmental Covariates of Sexual Behavior Problems among Youth Referred to Child Protection Services Supplemental Material, sj-pdf-1-sax-10.1177_10790632211047184 for A Longitudinal Examination of Developmental Covariates of Sexual Behavior Problems among Youth Referred to Child Protection Services by Stéphanie Chouinard-Thivierge Patrick Lussier and Isabelle V. Daignault in Sexual Abuse: A Journal of Research and Treatment

## Notes

1 Using descriptive information about each case (see Table 1), we determined whether a regression model could identify cases selected for the present study. For the childhood-onset group, 22.7% of the cases in the study were correctly identified by the regression analyses (Nagelkerke’s pseudo R^2^ = .11), with only one factor significantly contributing to the model (total number of CPS referrals for any reasons; (*B* = .063 (.035), *p* = .074)). For the adolescence-onset group, 7.3% of cases included in the study were correctly identified by regression analyses (Nagelkerke’s pseudo R^2^ = .129), with only one factor contributing to the statistical model (age at first CPS referral; (*B* = 1.09 (.241), *p* < .001)).

2 First, this analysis is not a purely statistical approach to the issue of the covariates of SBP. The authors preselected the covariates examined in the current study based on the scientific literature dealing with SBP. Second, only a circumscribed set of covariates —ACE —are examined, and their use is based on attachment theories of sexual offending. Therefore, the number of ACE examined was limited. Fourth, in the stepwise approach, the covariates were statistically examined in chronological order of appearance in a child’s life course. Entry into the model was prioritized based on the time of appearance of ACE, as these may have an effect on a child’s social and behavioral development.

3 Note that the statistical analyses and the findings reported in the study are based on a listwise deletion of cases due to missing data. The listwise approach was used because the amount of missing data across variables was small and not significant. The only indicator with significant missing information was “ethnicity” (14%). All models were examined and compared both with and without missing cases for the ethnicity indicator and none of the findings reported were affected. For simplicity, findings based on listwise deletion are reported.
