# Supplementary material for: A Longitudinal Examination of Developmental Covariates of Sexual Behavior Problems among Youth Referred to Child Protection Services
Source: Sex Abuse. 2021 Sep 30;34(5):537–67. doi: 10.1177/10790632211047184 (PMC9260481; doi:10.1177/10790632211047184)
Supplement: sj-pdf-1-sax-10.1177_10790632211047184 – Supplemental Material for A Longitudinal Examination of Developmental Covariates of Sexual Behavior Problems among Youth Referred to Child Protection Services [file sj-pdf-1-sax-10.1177_10790632211047184.pdf]

SupplementalTables.SA-20-07-066.R1 – Supplemental material for A Longitudinal Examination of Developmental Covariates of Sexual Behavior Problems among Youth Referred to Child Protection Services

Description : Supplemental material (File : SupplementalTables.SA-20-07-066.R1) for A Longitudinal Examination of Developmental Covariates of Sexual Behavior Problems among Youth Referred to Child Protection Services by Stéphanie Chouinard-Thivierge, Patrick Lussier, and Isabelle V. Daignault in Sexual Abuse journal.

Supplemental Table 4

Stepwise logistic regression analyses of the developmental covariates for the childhood-onset of sexual behavior problems

	Unweighted models			Weighted models		
Covariates	Model 1<br>Infancy<br>OR (95% C.I.)	Model 2<br>Early childhood<br>OR (95% C.I.)	Model 3<br>Late childhood<br>OR (95% C.I.)	Model 1<br>Infancy<br>OR (95% C.I.)	Model 2<br>Early childhood<br>OR (95% C.I.)	Model 3<br>Late childhood<br>OR (95% C.I.)
Infancy						
None	-	-	-	-	-	-
Early childhood						
Psychological abuse	-	3.60 (1.25-10.35)*	3.23 (.94-11.17)	-	3.63 (1.25-10.35)***	3.12 (1.15-8.49)*
Late childhood						
Parental long-term absence	-	-	.49 (.26-.93)*	-	-	.47 (.27-.83)**
Psychological abuse	-	-	2.21 (1.07-4.57)*	-	-	2.01 (1.09-3.71)*
Delinquency	-	-	5.43 (2.83-10.42)***	-	-	5.59 (3.23-9.67)***
Persistent aggression	-	-	2.62 (.99-6.89)*	-	-	2.49 (1.11-5.58)*
Control variables						
Sex	2.75 (1.59-4.37)***	2.56 (1.48-4.43)**	1.92 (1.05-3.54)*	2.85 (1.75-4.64)***	2.62 (1.60-4.29)***	2.05 (1.19-3.53)*
Ethnicity	.38 (.11-1.26)	.37 (.11-1.27)	.37 (.09-1.39)	.37 (.21-1.11)	.34 (.11-1.07)	.38 (.11-1.25)
Early age at 1st CPS referral	.91 (.51-1.65)	.82 (.45-1.5)	.75 (.37-1.52)	.88 (.53-1.47)	.79 (.47-1.35)	.73 (.39-1.34)
Siblings	.87 (.72-1.03)	.85 (.71-1.02)	.82 (.67-.99)*	.87 (.74-1.01)	.85 (.72-.99)*	.80 (.76-.96)*
Parental criminal record	1.13 (.64-1.97)	1.07 (.60-1.89)	.79 (.41-1.53)	1.10 (.67-1.78)	1.03 (.62-1.69)	.82 (.47-1.44)
Number of CPS referrals	1.07 (1.02-1.12)*	1.05 (.99-1.10)	1.00 (.94-1.07)	1.07 (1.03-1.12)*	1.05 (1.01-1.10)*	1.01 (.96-1.07)
Constant	.38**	.46*	.50	.19***	.23***	.25***
Model fit						
Nagelkerke Pseudo R^2	.06	.08	.20	.06	.08	.20
Model coefficients X^2 (df)	24.23 (6)***	30.53 (7)***	79.87 (11)***	32.83 (6)***	41.99 (7)***	109.75 (11)***
Block coefficients X^2 (df)	N/A	6.30(1)*	49.34 (4)***	N/A	9.16 (1)*	67.76 (4)***

Note. *Sample size for these analyses is 285. CI = confidence interval; OR = odds ratio.*
**p < .05; **p < .01; ***p < .001*

Supplemental Table 6

Stepwise logistic regression analyses of the developmental covariates for the continuity of sexual behavior problems

	Unweighted models			Weighted models		
Covariates	Model 1 Infancy OR (95% C.I.)	Model 2 Early childhood OR (95% C.I.)	Model 3 Late childhood OR (95% C.I.)	Model 1 Infancy OR (95% C.I.)	Model 2 Early childhood OR (95% C.I.)	Model 3 Late childhood OR (95% C.I.)
Infancy						
Parental drug/alcohol abuse	.16 (.04-.59)**	.16 (.04-.59)**	.19 (.05-.76)*	.15 (.05-.46)**	.34 (.09-1.21)	.24 (.06-.94)*
Early childhood						
Parental drug/alcohol abuse	-	-	-	-	.17 (.04-.82)*	.15 (.03-.79)*
Late childhood						
Parental Neglect	-	-	2.86 (1.07-7.63)*	-	-	-
Exposure to intimate partner violence	-	-	.32 (.11-.93)*	-	-	.30 (.11-.78)*
History of placement	-	-	-	-	-	2.74 (1.25-6.00)*
Control variables						
Sex	1.30 (.54-3.14)	1.30 (.54-3.14)	1.13 (.44-2.86)	1.29 (.59-1.57)	1.57 (.69-3.59)	1.41 (.60-3.35)
Ethnicity	1.67 (.13-21.43)	1.67 (.13-21.43)	2.41 (.16-33.37)	1.99 (.25-15.95)	1.86 (.24-14.64)	2.03 (.24-17.48)
Early age at 1st CPS referral	7.67 (2.45-23.96)***	7.67 (2.45-23.96)***	6.62 (2.06-21.27)**	8.01 (3.19-20.13)***	18.54 (4.94-69.52)***	14.91 (3.89-57.03)**
Siblings	.97 (.72-1.30)	.97 (.72-1.30)	.98 (.72-1.32)	.97 (.74-1.26)	.98 (.76-1.28)	.96 (.73-1.26)
Parental criminal record	2.24 (.94-5.31)	2.24 (.94-5.31)	2.62 (.99-6.93)	2.16 (1.06-4.41)*	2.29 (1.12-4.70)*	2.68 (1.17-6.16)*
Number of CPS referrals	.99 (.93-1.06)	.99 (.93-1.06)	.98 (.90-1.06)	.99 (.94-1.05)	.99 (.95-1.06)	1.01 (.95-1.08)
Constant	.54	.54	.29***	.26**	.21**	.20**
Model fit						
Nagelkerke Pseudo R^2	.12	.12	.17	.13	.15	.19
Model coefficients X^2 (df)	22.71 (7)**	22.71 (7)**	79.87 (11)***	33.06 (7)***	38.65 (8)***	50.73 (10)***
Block coefficients X^2 (df)	8.43 (1)**	8.43 (1)**	49.34 (4)***	12.87 (1)***	5.59 (1)*	12.08 (2)***

Note. *Sample size for these analyses is 138. CI = confidence interval; OR = odds ratio.*
**p < .05; **p < .01; ***p < .001*
